# Reduction of Glucocorticoid Receptor Function in Chronic Fatigue Syndrome

**DOI:** 10.1155/2018/3972104

**Published:** 2018-06-10

**Authors:** Megan Lynn, Laura Maclachlan, Andreas Finkelmeyer, James Clark, James Locke, Stephen Todryk, Wan-Fai Ng, Julia L. Newton, Stuart Watson

**Affiliations:** ^1^Institute of Neuroscience, Newcastle University, Newcastle upon Tyne, UK; ^2^Department of Public Health and Community Medicine, University of Gothenburg, Gothenburg, Sweden; ^3^Institute of Cellular Medicine and NIHR Newcastle Biomedical Research Centre, Newcastle University, Newcastle upon Tyne, UK; ^4^Faculty of Health and Life Sciences, Northumbria University, Newcastle upon Tyne, UK; ^5^Newcastle Hospitals NHS Foundation Trust, Newcastle upon Tyne, UK; ^6^Northumberland, Tyne and Wear NHS Foundation Trust, Newcastle upon Tyne, UK

## Abstract

Glucocorticoid receptor (GR) function may have aetiopathogenic significance in chronic fatigue syndrome (CFS), via its essential role in mediating inflammatory responses as well as in hypothalamic-pituitary-adrenal axis regulation. GR function can be estimated ex vivo by measuring dexamethasone (dex) modulation of cytokine response to lipopolysaccharide (LPS), and *in vivo* using the impact of dex on cortisol levels. This study aimed to compare the GR function between CFS (*n* = 48), primary Sjögren's syndrome (a disease group control) (*n* = 27), and sedentary healthy controls (HCs) (*n* = 20), and to investigate its relationship with clinical measures. In the GR ex vivo response assay, whole blood was diluted and incubated with LPS (to stimulate cytokine production), with or without 10 or 100 nanomolar concentrations of dex. Cytometric bead array (CBA) and flow cytometry enabled quantification of cytokine levels (TNF*α*, interleukin- (IL-) 6, and IL-10) in the supernatants. In the *in vivo* response assay, five plasma samples were taken for determination of total cortisol concentration using ELISA at half-hourly intervals on two consecutive mornings separated by ingestion of 0.5 mg of dex at 11 pm. The association of the data from the *in vivo* and ex vivo analyses with reported childhood adversity was also examined. CFS patients had reduced LPS-induced IL-6 and TNF*α* production compared to both control groups and reduced suppression of TNF*α* by the higher dose of dex compared to HCs. Cortisol levels, before or after dex, did not differ between CFS and HCs. Cortisol levels were more variable in CFS than HCs. In the combined group (CFS plus HC), cortisol concentrations positively and ex vivo GR function (determined by dex-mediated suppression of IL-10) negatively correlated with childhood adversity score. The results do not support the hypothesis that GR dysregulation is aetiopathogenic in CFS and suggest that current and future endocrine cross-sectional studies in CFS may be vulnerable to the confounding influence of childhood trauma which is likely increased by comorbid depression.

## 1. Introduction

Chronic fatigue syndrome (CFS) has a prevalence of 2% in the UK [1, 2]. It is defined by profound, persistent, medically unexplained fatigue lasting at least 6 months, which is not caused by ongoing exertion, not significantly eased by rest, and is severe enough to cause considerable loss of function [3–5]. Alongside this are symptoms of inflammation, pain, cognitive deficits, and psychiatric and bowel problems [4]. Often, biological tests and physical examinations are unremarkable. CFS affects all ages and the peak age of onset is 20–40. Full recovery is rare [2, 6] and comorbidity with depression is common.

Many putative causes of CFS have been investigated but the absence of an agreed pathogenesis impacts the development of effective diagnostics and treatments. It is likely that multiple factors contribute which involve a number of interacting biological, environmental, and psychosocial factors [1, 2, 5, 7–11].

The recognised temporal relationship between stressors and the onset and course of CFS suggests an aetiopathogenic role for systems controlling the stress response including the sympathetic nervous system and the hypothalamic-pituitary-adrenal (HPA) axis [4, 8, 12–17]. The functionality of the glucocorticoid receptor (GR), determined by its sensitivity, affinity, and density and by its interaction with transcription factors [18], is arguably the defining factor in HPA axis regulation [19] and responsible, in a large part, for basal concentrations of cortisol throughout the day. In HPA axis downregulation in response to the GR agonist, dexamethasone (dex) is the most commonly used *in vivo* method of determining GR function. An ex vivo technique dependent on the inhibitory effect of GR activation on cytokine release is also utilized [20, 21]. Cross-sectional studies in CFS tend to show basal hypocortisolaemia [22, 23], attenuated diurnal variation [4, 24], an attenuated response to activation by CRH or ACTH [16, 23, 25–28], an enhanced suppression by dex [10, 17, 28–30], and an enhanced dex-induced suppression of IL-6, TNF*α*, IL-10, and IL-4 synthesis [21, 31, 32] and of peripheral blood mononuclear cell proliferation [18]. Genetic studies in CFS have shown the salience of functional single nuclear polymorphisms in NR3C1 [31, 32], which codes for GR, and have also shown hypomethylation of the 1F promotor region of this gene [33–35]. The endocrine findings however are not consistent, and elevated [36] or normal [18, 27, 25, 37] basal cortisol levels have also been reported, as has a normal cortisol response to wakening [25] and to psychosocial stress [21, 38]. There is also some evidence of an association of HPA axis dysregulation with symptom severity and poorer prognosis [15, 39] but only speculation about the mechanism through which HPA axis abnormalities may result in the symptoms of CFS; glucose supply [40], hypotension with associated reduced cerebral perfusion [41], and CRH-induced appetite and sleep disturbance have been considered [16].

The HPA axis interacts with many other systems, notably the immune system [42]. Glucocorticoids (GCs) modulate immune responses by altering gene expression, transcription, translation, and protein secretion [42], either directly, by decreasing transcription of the genes which code for cytokines, or indirectly, by inhibiting proinflammatory transcription factors [42]. GCs inhibit, with different sensitivities, cytokines such as IL-6, IL-1, and TNF (TNF the most, IL-6 the least) [42, 43]. Immune activation, with an increase in proinflammatory cytokine concentrations, including IL-6, TNF*α*, and IL-1 [2, 44, 45], has been rather inconsistently, for example [32], demonstrated in CFS, may be secondary to insufficient glucocorticoid signaling [7], and may result in pain, fatigue, cognitive deficits, and other symptoms which are characteristic of CFS [12, 16, 38, 46].

In order to examine the nature, extent, and impact of HPA axis dysregulation in CFS, we sought to compare GR function using both *in vivo* and ex vivo assessment. We therefore examined the HPA axis and immune system function in a sample of patients with CFS and in healthy comparators and in participants with the systemic autoimmune condition, primary Sjögren's syndrome (pSS), who acted as disease group comparators.

## 2. Methods

### 2.1. Participants

Three groups were recruited. The study was carried out in accordance with the Declaration of Helsinki. The study design was approved by the Newcastle and North Tyneside Ethics Committee. All participants provided written informed consent. Participants were aged 22–68 years old. Exclusion criteria consisted of age < 18 years, a current or past axis I psychiatric diagnosis confirmed using the Structured Clinical Interview for DSM-IV [47, 48], and use, in the 72 hours prior to enrolment, of antihypertensives, antidepressants, or analgesics. Samples were collected as part of an MRC-funded cohort study (MRC MR/J002712/1). 48 participants with CFS (13 males (mean age 52.2) and 35 females (mean age = 44.9)) were recruited via the local CFS clinical service, all fulfilled the Fukuda diagnostic criteria, and had a mean FIS of 88 and CTQ of 32. Twenty healthy comparators (HC; 7 males (mean age = 43.1) and 13 females (mean age 44.9)) were recruited from a HC database, word of mouth, social media, and advertisement in the hospital (mean FIS = 4, CTQ = 29). HCs were age and sex matched to the patients, and attempts were made to match on activity levels using the Mean International Physical Activity Questionnaire (IPAQ) although the mean CFS IPAQ rating was “low” and the HC rating was “medium.” Primary Sjögren's syndrome (pSS) patients (*n* = 27) fulfilled the American European Consensus Group classification [49] and were recruited from the United Kingdom pSS Registry [50].

### 2.2. Symptom Assessment Tools

The CFS participants completed the Fatigue Impact Scale (FIS) [51] and the Childhood Trauma Questionnaire-Short Form (CTQ-SF) [52]. The FIS quantifies individual perception of the impact that fatigue has on daily functioning (Fisk et al.) [53]. There are 40 items, each scored on a 5-point Likert scale providing a continuous scale of 0–160. It comprises three subscales looking at the impact that fatigue has on physical (10 items: motivation, effort, stamina, and coordination), psychosocial (20 items: isolation, emotions, coping, and workload), and cognitive (10 items: concentration, memory, and thinking) functioning. A higher score indicates greater fatigue. The CTQ is a 28 item self-report scale which measures the frequency and severity of childhood adversity. It consists of five factors: emotional abuse, physical abuse, sexual abuse, emotional neglect, and physical neglect, and possesses good psychometric properties [52]. Items are scored on a Likert scale, with responses ranging from 1 (“never true”) to 5 (“very often true”).

### 2.3. *In Vivo* Assessment of HPA Axis Function by Measurement of Cortisol Levels in Response to Low-Dose dex in CFS Compared to Healthy Controls

In the 48 CFS patients and the first 10 healthy controls (HCs), plasma samples were taken in lithium-heparin vacutainers at 30-minute intervals between 10 am and noon on two consecutive days (day 1 and day 2). At 11 pm on day 1, participants took oral dex (0.5 mg). Practical consideration meant that only the baseline blood (10 am day 1 sample) were taken for the other 10 HCs. Within one hour of collection, the blood was spun at 1600 g for 10 minutes at room temperature. Aliquots of plasma were extracted and stored at −80°C until analysis. Plasma cortisol concentrations were quantified using 15 lot-matched cortisol ELISA kits, supplied by Abcam and used according to the manufacturer's protocol. The lower limit of cortisol detection was 2.44 ng/ml.

### 2.4. Ex Vivo Measurement of GR Function: The Glucocorticoid Receptor Response Assay

The GR response assay [20] utilised lithium-heparin-treated blood taken at 10 am on day 1. It was set up in sterile 48-well plates within 3 hours of collection. Blood was diluted 1/10 with room temperature RPMI 1640 containing penicillin-streptomycin and L-glutamine, mixed thoroughly by inversion, and then incubated for 24 hours at 37°C in a humidified atmosphere of 95% air and 5% CO_2_. There were four conditions, “null” (medium alone), “LPS” in which 50 *μ*l of a 200 *μ*g/ml LPS solution was added to the 400 *μ*l of diluted blood (to stimulate cytokine production from cells), “dex10” in which 50 *μ*l of a 100 nanomolar (nM) dex solution was also added, and “dex100” in which a tenfold stronger dex solution was added. After incubation, the assay plates were spun in a 4°C centrifuge for 10 minutes at 1000 RPM. 300 *μ*l supernatant samples were then harvested for each condition, transferred to 0.6 ml microcentrifuge tubes, and stored at −20°C. Cytokine concentrations (TNF*α*, IL-6, and IL-10) were determined using Cytometric Bead Array (CBA) and flow cytometry according to the manufacturer's instruction (BD Bioscience). Percentage suppression on dex 10 nM (% dex10) and on dex 100 nM (% dex100) was calculated using the following equation:

(1) $$\begin{array}{c} \%\;cytokine\;suppression=100-\left(\frac{raw\;cytokine\;level\;on\;dex10\;nM\text{ or }on\;dex100\;nM}{raw\;LPS‐stimulated\;cytokine\;level}\times 100\right). \end{array}$$

### 2.5. Baseline Cytokine Levels

Gel-based specimen tubes were used to collect a further serum sample at 10 am on day 1 for baseline measurement of a range of inflammatory markers. These were spun within 3 hours at 1600 RPM for 10 minutes at room temperature. 2 × 1 ml of serum was extracted and stored at −80°C until analysis which utilized the method described above, that is, CBA and flow cytometry. Two different dilutions of serum samples were required for the measurement of different cytokines; this was therefore conducted in 2 batches for CFS (*n* = 45), HC (*n* = 19), and pSS (*n* = 9) participants.

### 2.6. Data and Statistical Analysis

Statistical tests were carried out using SPSS version 23 and the “R” statistical package. Graphs were produced using GraphPad Prism version 5.01 and MATLAB. *p* values are two-tailed with significance set at *p* < 0.05.

Repeated measures ANOVA was conducted with time (5) as within and group (2) as between factors to examine cortisol concentrations on each of the two days. Area under the curve (AUC) was also calculated using trapezoid integration, both for day 1 and day 2. Specifically, AUC with respect to ground (AUCg), considered to be a measure of overall cortisol output including baseline activity, and AUC increase (AUCi), a putative measure of the sensitivity of GR to modulation, were calculated [54]. The difference between AUCg on the 2 days was also calculated (delta AUCg). Shapiro-Wilk and QQ plots (data not shown) revealed that even after Box-Cox transformation, neither AUC nor cytokine data met the assumptions required for ANOVA; thus, nonparametric comparisons were used. Spearman correlations were conducted to examine the relationship between childhood adversity and endocrine parameters. All data, shown or not shown, is available for scrutiny upon request.

## 3. Results

### 3.1. *In Vivo* Assessment of HPA Axis Function by Measurement of Cortisol Levels in Response to Low-Dose dex in CFS Compared to Healthy Controls

**Figure 1:**
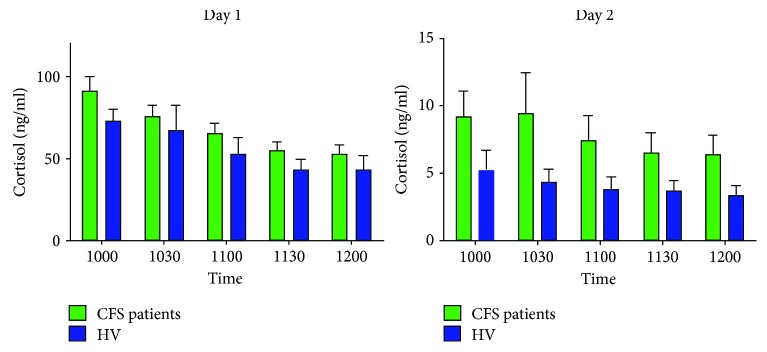
Total cortisol values at five time points over two days in participants with CFS and healthy controls. Bar chart showing cortisol concentrations in CFS patients and healthy controls at five morning time points over two consecutive days. Participants administered oral dex at 11 pm on day 1. Data are shown as mean plus standard error of the mean.

There was a significant effect of time on day one (*F* = 16.61, df = 4, 56, *p* < 0.0005) but not on day two (*F* = 0.87, df = 4, 56, *p* = 0.418). No effect of group on either day (day one, *F* = 0.73, df = 1, 56, *p* = 0.398; day 2, *F* = 0.79, df = 1, 56, *p* = 0.378) (see Figure 1). Cortisol AUCs (g or i, day one, day two, or delta) did not differ (*p* > 0.2).

### 3.2. Ex Vivo Assessment of HPA Axis Function: The Glucocorticoid Receptor (GR) Response Assay

GR response assay blood was not taken for two CFS patients. One HC was removed due to an abnormally high null value and one CFS patient removed due to not stimulating sufficiently on LPS. Analysis was therefore conducted on samples taken from CFS (*n* = 40), HC (*n* = 19), and pSS (*n* = 27) participants.

In the null sample, pSS participants had higher IL-10 levels than the CFS or HC participants but there were no differences between groups for IL-6 or TNF*α*. LPS induced a robust cytokine response, and after LPS, group differences were evident such that, for the positive cytokines IL-6 and TNF*α*, pSS participants had higher levels than CFS participants, who had higher levels than HCs. For IL-10, the difference was between pSS (higher) and HCs. Median cytokine levels were, on the whole, lower in the dex 10 nM samples and invariably in the dex 100 nM samples than the LPS alone samples. Percentage suppression was greater for samples incubated with dex 100 nM than those incubated with dex 10 nM. Percentage suppression with dex 10 nM or dex 100 nM was not different between the groups, except for a greater suppression in the dex 100 nM condition determined using TNF*α* (see Table 1).

**Figure 2:**
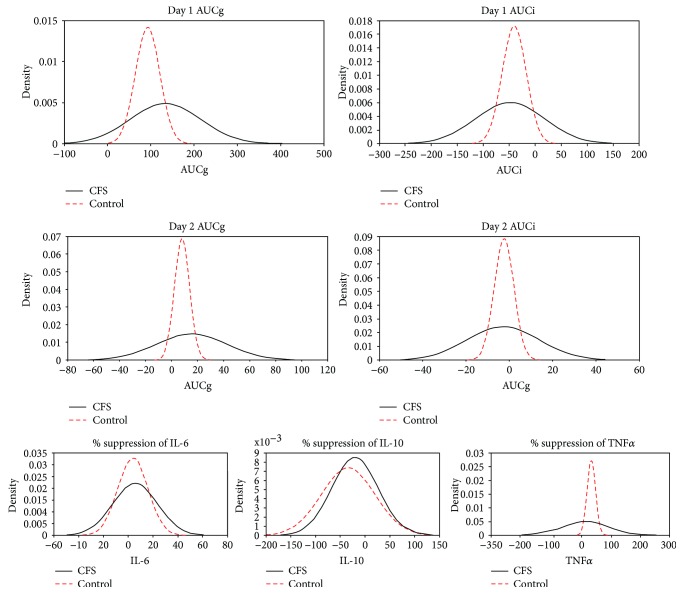
Frequency density graphs for cortisol area under the curve data and for the suppression of cytokine release by 10 nM solution of dexamethasone. Frequency density graphs for participants with chronic fatigue syndrome and healthy controls for area under the curve (AUC) with respect to ground (g) and increase (i) and for % suppression of TNF*α*, IL-6, and IL-10 by dex10.

Individual AUC and cytokine values were visualized using frequency density plots. Visual inspection suggested greater variability in patients compared to HCs with some patients showing less suppression (see Figure 2). In order to investigate this, we performed post hoc *t*-tests of standard deviations derived from Bayesian hierarchical models of outcome measures under Gaussian (normal) priors. The advantage of this approach lies in its treatment of parameters as sampling variables rather than population attributes which allows us to formally compare modelled estimates of their values. Significance was assessed by comparing the 95% credible intervals of the posterior distributions. Vague priors were used for the mean and standard deviation, and analysis was carried out using the BEST package in the R statistical environment [55]. Results are displayed in Table 1. There was no difference in standard deviation of day 1 AUCg distributions. There was a significant difference in standard deviations for day 1 AUCi (*p* = .014), day 2 AUCg (*p* < .0005), and day 2 AUCi (*p* < .0005). There was also a significant difference in standard deviations of TNF distributions (*p* = .001). The difference in IL-6 was marginal (*p* = .054) though there was no significant difference in IL-10 distributions (*p* = .774).

### 3.3. The Relationship between Reported Adversity and HPA Axis Function in CFS and Controls

Correlation coefficients, in CFS participants, reveal a negative relationship between the CTQ score (and the emotional subscores) and cortisol AUC but no significant relationship with % suppression (after incubation with 10 nm dex). In HCs, there was no significant relationship between CTQ scores and cortisol AUC, but there was a positive relationship with percentage IL-6 suppression (see Table 2). In the combined sample, of CFS and HC (see Table 3), the significant correlations were between the CTQ total score and AUCg (positive) and IL-10 (negative).

### 3.4. Baseline Cytokine Levels

**Figure 3:**
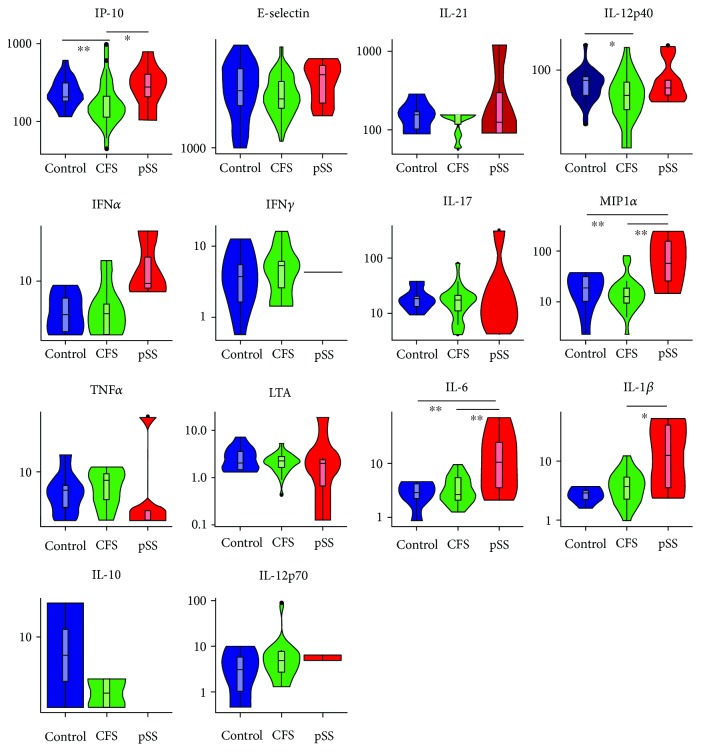
Violin plot showing baseline cytokine and chemokine concentrations. Baseline cytokine and chemokine concentrations (in pg/ml); asterisks refer to Mann–Whitney *U* comparisons ^∗∗^*p* < 0.01, ^∗^*p* < 0.05.

CFS patients showed reduced production of IP-10 and IL-12/23p40 compared to HC and of IP-10, MIP1*α*, IL-6, and IL-1*β* compared to pSS participants. pSS participants showed increased production of MIP1*α* and IL-6 compared to HV. A violin plot was designed post hoc using “R” statistical software to visualize variance between populations and determine whether subpopulations were present and is displayed in Figure 3.

## 4. Discussion

We did not demonstrate a difference in cortisol levels between participants with CFS and healthy volunteers. This differs from the majority of cross-sectional HPA axis studies in this population (see “Introduction” and [10] for review). This difference may be related to the population; the sample here, for instance, was rigorously screened for comorbid depression, and Papadopoulos et al. [36] have previously demonstrated that dex-induced cortisol suppression differed only in CFS patients with comorbid depression or it may be a type II error consequent on the small sample size combined with the marked variation in cortisol levels in CFS as highlighted by the frequency density graph and the significantly greater cortisol variability in patients with CFS. The aetiopathogenic relevance of this variability is unknown but it suggests a lack of precision in cortisol regulation [56]. The heterogeneity in cortisol concentrations may suggest clinical heterogeneity within the diagnostic grouping of CFS and emphasizes the impact of disparate and competing factors on GR function including current and previous stressors, the common use of antidepressants [20, 54] (even in those who have never met criteria for major depressive disorder) [20, 57], and the impact of a primary dysregulation of proinflammatory cytokines [58].

The baseline cytokine data emphasized the status of pSS as an inflammatory disorder. The ex vivo data revealed a reduced capacity for a proinflammatory cytokine response to LPS in CFS compared with HCs (and an increased responsivity compared to the pSS participants). It further revealed that (independent of group) incubation with dex, in a dose-dependent manner, as expected, suppressed cytokine release. The percent suppression of LPS-induced TNF*α* release by 100 nM solution of dex was less in CFS patients than HCs. This may be suggestive of reduced GR function in CFS but any such interpretation must be made with caution as the impact of 10 nM dex did not significantly differ between CFS and HCs, neither was a significant effect seen when IL-6 or IL-10 was used as the output variable. That TNF*α* was most sensitive to suppression by dex accords with the existing literature [42], is congruent with the theory that GCs may preferentially inhibit Th1 over Th2 cells [59], and suggests that TNF*α* may be the most appropriate cytokine for GR response assay studies in CFS. The variability in percentage suppression of cytokine levels by dex is also greater in CFS than healthy or pSS controls.

In CFS participants, there was a relationship between the score on the childhood trauma questionnaire and cortisol AUCs such that higher reported levels of early adversity correlated negatively with cortisol. Interestingly, a different pattern was seen in healthy volunteers in whom reported childhood adversity associated positively with dex-induced IL-6 suppression in the absence of an effect of cortisol concentrations. When the groups were combined to maximise power, a negative relationship between reported adversity and cortisol levels and a positive relationship with GR function (here shown using IL-10 not IL-6) are revealed.

The variability in cortisol has implications for the interpretation of existing and future endocrine cohort studies in CFS because of the associated risk of type I and type II errors; our data, for instance, would suggest that the proportion of participants in a sample who experienced childhood adversity will be expected to determine the likelihood that basal hypocortisolaemia will be shown. In addition to the CTQ total score, we report here also the emotional neglect subscale, having previously argued that the pervasive nature of emotional neglect ensures that it enacts the greater sustained impact on behavioural and endocrine function [60, 61].

There can be few who argue with the notion that, in the general population, early adversity, acting for instance through methylation or other epigenetic mechanisms, impacts GR function and so GR mediated negative feedback on the HPA axis and thus cortisol synthesis [62, 63] and, further, that this has relevance for understanding the pathophysiology of mood disorders [64]. It is of interest, here, to consider the implication that this has for our understanding of the pathophysiology of CFS and for the interpretation of endocrine studies in this population. We have previously postulated that childhood adversity is not a risk factor for CFS per se, but it can appear to be because of the impact of comorbid or misdiagnosed depression [65]. It has been further conjectured that comorbid depression may commonly confound CFS studies [36] and, just as this may lead to erroneous finding of increased rates of childhood adversity in CFS, similarly, it may explain the methylation pattern [66] including in the NR3C1-1F promoter region [33], the increased GR function (shown using the DST, the dex/CRH test [28], or ex vivo measures), and the basal hypocortisolaemia which have been (inconsistently [10]) shown in previous CFS studies. In this current study, CTQ scores were not greater in the CFS participants than HCs, and it is interesting to note that the basal cortisol or GR function as determined by post-dex cortisol or dex-induced suppression of cytokine synthesis was not convincingly different.

Despite our rigorous exclusion of those who met the diagnostic criteria for depression and the lack of difference in childhood adversity reported by CFS patients compared with HCs, there was a signal that HPA axis regulation was different in CFS; the variability of pre- and post-dex cortisol levels and of dex-induced cytokine suppression was increased in CFS, the proinflammatory cytokine response to LPS was attenuated, and the TNF*α* suppression by the larger dex dose was greater, and, whilst we do not want to make too much of this, the graph suggested (but the stats did not back up) the possibility that post-dex cortisol was lower in CFS than HCs. Further research is needed to understand the cause and significance of this data; this will need large, well-characterised groups and will need consideration to be given to the interacting networks of biological, psychological, and social factors.

## Figures and Tables

**Table 1:** Standard deviations for endocrine data between chronic fatigue and healthy participants.

	CFS	Healthy controls	Difference	*p* value
Day 1 AUCg	79.4 (63.1, 97.5)	72.1 (38.3, 113.2)	7.32 (−38.8, 48.4)	0.389
Day 1 AUCi	66.3 (52.9, 81.0)	27.5 (14.9, 43.4)	38.75 (16.9, 60.3)	0.014
Day 2 AUCg	25.4 (20.0, 31.4)	6.8 (3.7, 10.8)	18.61 (11.6, 25.8)	<0.0005
Day 2 AUCi	16.1 (12.7, 19.7)	5.3 (2.8, 8.4)	10.75 (5.9, 15.5)	<0.0005
% suppression TNF*α*	31.2 (24.3, 39.0)	15.6 (10.5, 21.3)	15.6 (6.1, 25.1)	0.001
% suppression IL-6	18.3 (14.2, 22.9)	13.0 (8.9, 17.9)	5.3 (−1.2, 11.8)	0.054
% suppression IL-10	47.4 (36.5, 59.3)	56.4 (38.2, 78.0)	−9.1 (−33.8, 13.9)	0.774

AUCg: area under the curve with respect to ground; AUCi: area under the curve with respect to increase. The % suppressions refer to the percentage difference in cytokine concentration in the dexamethasone 10 nM condition compared with the LPS condition. Values presented are the mean values of the posterior distribution of standard deviations with 95% credible intervals in brackets. Differences are significant if the credible interval does not include zero. *p* values refer to the proportion of the posterior mean standard deviation difference plot which is less than or equal to zero.

**Table 2:** Ex vivo glucocorticoid receptor response assay data.

		HC (*n* = 19)	CFS (*n* = 40)	PSS (*n* = 27)	KW	CFS versus HC	CFS versus PSS	PSS versus HC
IL-6	Null	1.3 (0.4 to 2.3)	1.6 (0.7–5.5)	1.3 (0.8 to1.7)	0.193	0.115	0.158	0.841
	LPS	14515.2 (12521.4 to 15196.4)	9517.8 (6994.4 to 12705.7)	16407.6 (12750.4 to 21912.2)	**<0.0005**	**0.001**	**<0.0005**	0.111
	dex10	13070.1 (10768 to 15175.2)	9013.1 (6268.4 to 12568.1)	15517.0 (12445.0 to 20557.3)	**<0.0005**	**0.007**	**<0.0005**	0.096
	dex100	7035.9 (5553.0 to 10618.0)	5606.2 (3394.6 to 7914.2)	10757.4 (7879.5 to 12655.4)	**<0.0005**	**0.037**	**<0.0005**	**0.014**
	% suppression dex10	3.0 (−4.5 to 12.1)	7.1 (−3.6 to 15.2)	4.3 (−1.1 to 11.8)	0.806	0.626	0.561	0.938
	% suppression dex100	47.1 (24.4 to 54.1)	40.5 (28.4 to 54.8)	36.2 (24.7 to 48.9)	0.235	0.697	0.172	0.113
	LPS versus dex10	0.171	**0.019**	**0.008**				
	LPS versus dex100	**<0.0005**	**<0.0005**	**<0.0005**				
	% suppression dex10 versus dex100	**<0.0005**	**<0.0005**	**<0.0005**				

IL-10	Null	0.0 (0.0 to 0.0)	0.0 (0.0 to 0.5)	0.4 (0.0 to 1.0)	**0.007**	0.615	**0.003**	**0.031**
	LPS	121.7 (66.6 to 260.0)	95.8 (58.3 to 149.5)	149.7 (80.3 to 240.6)	**0.032**	0.114	**0.011**	0.585
	dex10	179.3 (100 to 264.3)	111.9 (76.7 to 151.8)	157.7 (98.0 to 249.0)	**0.029**	**0.020**	**0.040**	0.772
	dex100	248.1 (144.7 to 314.6)	135.7 (81.4 to 214.0)	197.6 (115.2 to 250.9)	**0.004**	**0.003**	**0.014**	0.346
	% suppression dex10	−17.5 (−59.4 to −1.0)	−5.3 (−31.6 to 6.4)	−12.2 (−23.6 to 9.8)	0.375	0.249	0.779	0.177
	% suppression dex100	−56.1 (−110.4 to −24.5)	−29.2 (−95.2 to 1.9)	−33.7 (−69.6 to 4.1)	0.321	0.168	1.000	0.183
	LPS versus dex10	**0.027**	**0.010**	0.091				
	LPS versus dex100	**0.002**	**0.002**	**0.006**				
	% suppression dex10 versus dex100	**0.001**	**0.007**	**0.003**				

TNF*α*	Null	0.6 (0.0 to 1.2)	0.5 (0.0 to 0.9)	0.7 (0.0 to 1.0)	0.547	0.596	0.264	0.778
	LPS	1682.6 (895.0 to 2055.8)	937.5 (636.4 to 1262.9)	1872.4 (1275.1 to 2410.9)	**<0.0005**	**0.008**	**<0.0005**	0.403
	dex10	1072.0 (659.0 to 1473.4)	743.5 (500.0 to 900.2)	1428.2 (797.5 to 1942.8)	**0.001**	**0.019**	**0.001**	0.170
	dex100	261.4 (129.3 to 500.6)	246.2 (152.2 to 245.4)	389.2 (242.3 to 611.9)	**0.026**	0.511	**0.006**	0.129
	% suppression dex10	29.7 (25.0 to 37.2)	21.3 (10.6 to 37.5)	28.9 (12.7 to 39.9)	0.443	0.183	0.645	0.496
	% suppression dex100	82.7 (77.9 to 87.0)	74.9 (62.6 to 83.6)	76.9 (67.6 to 84.5)	0.080	**0.034**	0.818	0.060
	LPS versus dex10	**<0.0005**	**<0.0005**	**0.001**				
	LPS versus dex100	<0.0005	<0.0005	<0.0005				
	% suppression dex10 versus dex100	<0.0005	<0.0005	<0.0005				

Cytokine concentrations (in pg/ml) and percent cytokine suppression on 10 nm dexamethasone and 100 nm dexamethasone in healthy controls (HCs), participants with chronic fatigue syndrome (CFS), and primary Sjögren's syndrome (PSS). Comparisons are the *p* values for the independent samples Kruskal-Wallis (KW) comparison of the medians across the 3 groups (HC, CFS and PSS) and the Mann–Whitney U comparisons between two groups (e.g. HC versus CFS). The significance levels for the comparison of cytokine levels in the samples treated with LPS and those treated in addition with dexamethasone, using Related Samples Wilcoxon Signed Rank Test, are also shown for the three groups.

**Table 3:** Correlation coefficients for the relationship between childhood trauma and endocrine variables.

	CFS				HC				Combined			
	AUCg day 1	IL-6% suppression dex10	IL-10% suppression dex10	TNF*α* % suppression dex10	AUCg day 1	IL-6% suppression dex10	IL-10% suppression dex10	TNF*α* % suppression dex10	AUCg day 1	IL-6% suppression dex10	IL-10% suppression dex10	TNF*α* % suppression dex10
CTQ total score	**−0.40** ^∗∗^	0.03	0.24	−0.23	0.15	**0.64** ^∗∗^	0.44	0.02	−**0.32**^∗^	0.17	**0.29** ^∗^	−0.19
CTQ emotional neglect	−**0.33**^∗^	0.07	0.29	−0.22	0.20	**0.72** ^∗∗^	**0.53** ^∗^	−0.08	−0.21	0.22	**0.37** ^∗∗^	−0.17

Spearman's rho values with significance level indicated by asterisk (*p*^∗∗^ < 0.005, ^∗^ < 0.05) for relationship between childhood adversity and endocrine data in participants with CFS, healthy controls and in a combined group. Data analyses for each group are in the 4 columns below CFS, HC and Combined.

## Data Availability

The data used to support the findings of this study are available from the corresponding author upon request.
